# A Longitudinal dataset of sector-level patent data for Europe

**DOI:** 10.1016/j.dib.2025.112095

**Published:** 2025-09-22

**Authors:** P. Dimas, D. Stamopoulos, A. Protogerou

**Affiliations:** a Laboratory of Industrial and Energy Economics (LIEE), National Technical University of Athens (NTUA), 9 Heroon Polytechniou str., Zografou Campus, Athens 15780, Greece; b Department of Financial and Management Engineering, University of the Aegean, 41 Kountouriotou str., 82132, Chios, Greece

**Keywords:** Innovation, R&D, Sector data, International patent classification

## Abstract

This paper presents a comprehensive dataset of patent applications and grants at the 2-digit ISIC Rev.4/NACE Rev.2 sector level for a set of 24 European countries from 1985 to 2020. The data are assigned to sectors by applying weights of sector-technology field correspondence to European Patent Office (EPO) patent data retrieved from OECD. The dataset provides patent data based on the applicant’s and inventor’s country of residence, on an annual basis, in two formats: i) patent applications and grants at priority dates (flows), and ii) patent applications and grants stocks, estimated through the perpetual inventory method (PIM) using a 15 % deprecation rate. This unique dataset is suitable for sector/industry-level empirical research in the economic analysis of technology and R&D, indicatively in the fields of economics of innovation, international economics and industrial organization. Furthermore, it serves as a valuable resource for economic policy studies, especially in areas related to industry and science, technology, and innovation (STI) policy research.

Specifications Table

**Table:** 

Subject:	Social Sciences
Specific subject area	• Economics – Economics of Innovation; International Economics; Industrial Organization; Industrial Policy; Science Technology and Innovation (STI) Policy • Technology Management – R&D Management
Type of data	Tables
Data collection	Primary data about patent applications and grants (at priority dates) to/from the European Patent Office (EPO) by inventor’s and applicant’s country of residence (fractional counts applied for patents with multiple inventors/applicants), year, and technology field based on the International Patent Classification (IPC) fields were retrieved from OECD. Data about correspondence weights between ISIC Rev.4/NACE Rev.2 sectors (2-digit level) and IPC fields (1-digit level) were retrieved from the supplementary material of Lybbert & Zolas [1]
Data source location	1. Patent applications and grants to/from the EPO by applicant’s/inventor’s country of residence, year, and technology domains from OECD available at: https://data-explorer.oecd.org/ through the query: Patents by technology 2. Sector-technology field correspondence weights retrieved from Lybbert and Zolas' [1] supplementary material at^1^: https://sites.google.com/site/nikolaszolas/PatentCrosswalk?authuser=0
Data accessibility	Repository name: Mendeley Data Data identification number: 10.17632/fvhmttgc3s.2 Direct URL to data: https://data.mendeley.com/datasets/fvhmttgc3s/2
Related research article	None

## 1 Value of the Data

• A readily-available sector level dataset with extensive coverage across countries and economic sectors, spanning a long timeframe, that provides patent data (applications/grants, stocks/flows) that can be used to proxy technological innovation and measure innovation performance at both the sector and the country level across Europe.
• The dataset is developed using EPO patent data organized in IPC technology fields, which are not currently available from OECD, thus rendering it -to the best of our knowledge- a unique source of publicly available, sector-level patent data for Europe.
• In terms of coverage, the dataset offers a rich body of data spanning multiple sectors, ranging from usual patent applicants (e.g., pharmaceuticals, electronics), to low-tech sectors (e.g., textiles, paper and paper products) and non-industrial (service) sectors that can be engaged in technological innovation activities (e.g., telecommunications, computer programming, scientific research and development). In addition, it covers both EU and non-EU countries, including European global innovation leaders Norway, Switzerland, and the UK.
• The dataset is designed to support a broad spectrum of empirical applications and can accommodate academics, researchers, policy analysts and policymakers, industry professionals and other parties interested in the intersection of technology, innovation, economics, and industry/sector dynamics. Potential applications of the dataset include: 1) descriptive and comparative analyses at the sector and country level, 2) sector and/or country level case studies, 3) econometric and other statistical applications.
• The sector dimension is fully compatible with other sector-level economic data retrieved from major open-access data sources, such as Eurostat and OECD, rendering the dataset a valuable research asset. Characteristic use cases with significant implications for both practitioners and policymakers/analysts include: 1) growth accounting and investigation of the link between innovation intensity and productivity (labour, total factor) growth, 2) empirical applications of knowledge generation functions, where patent data can serve as (technological) innovation output, and 3) revealed comparative (technological) advantage calculations for innovative output, which can be linked to export performance (e.g., export market shares).

## 2 Background

Patent statistics have been at the forefront of empirical research as suitable proxies to track technological change, diffusion, and innovation, and have been frequently used as economic indicators in studies addressing the contribution of technology and innovation to economic growth and competitiveness at the firm and country level. This data paper aims to contribute to this ever-expanding literature by utilizing the Algorithmic Links with Probabilities (ALP) approach of Lybbert and Zolas [1] to offer a *meso*/sector-level dataset of patent applications and grants for 24 European economies during the period 1985–2020. Data regarding patent applications and grants to the EPO by inventor’s and applicant’s country of residence are drawn from OECD’s patent database are matched to ISIC Rev.4/NACE Rev.2 economic activities by correspondence weights, which are estimated through an ALP approach that uses keywords from sector descriptions to mine patent data and match them to corresponding sectors through a probabilistic framework [1]. In addition, we apply the perpetual inventory method (PIM) (Eq.1) to the matched patent data to develop stock measures of patent applications and grants that account for each sector’s inventive capacity, knowledge base, and technological competence:

(1) $$\begin{array}{c} S_{i,c,t}=P_{i,c,t}+\left(1-\delta \right)S_{i,c,t-1} \end{array}$$

where $S_{i,c,t}$ corresponds to patent applications/grants stock of sector i in country c at time t, $P_{i,c,t}$ is the number of patent applications/grants (flow) of sector i in country c at time t, and δ is a deprecation rate set at 15%, following the guidelines of the corresponding literature on patent statistics as innovation indicators. [2,3]

The dataset offers readily available sector level data that can be used in the economic analysis of technology and innovation, international trade and global value chains, and can also contribute to policy research on topics related to industrial policy and strategy, science, technology, and innovation (STI) policies, and competitiveness.

## 3 Data Description

The dataset [4] is organized in an excel file comprising six (6) sheets. Sheet 1 (info) contains important information about the background, structure, and content of the dataset. Sheet 2 (coverage) includes two tables that provide information on the sectors and countries covered. Sheets 3 to 6 include the main indicators of the dataset:

• Patent flows, organized by year, country, and sector, and accounting for patent type (applications and grants based on priority dates) and reference country (inventor’s or applicant’s country of residence).
• Patent stocks, organized in an identical format to patent flows and calculated through the PIM method.

In detail, Sheets 3 (flows_wide) and 4 (stock_wide) provide the two indicators in a wide (table) format where the year dimension servers as column headers, while Sheets 4 (flows_long) and 5 (stock_long) provide the data in a longitudinal format. The dataset is also available in a decomposed form of four (4) files (flows_wide, stock_wide, flows_long, and stock_long - one per spreadsheet) to accommodate direct imports into statistical software packages.

The information regarding the indicators’ dimensions is presented in Table 1:

**Table 1:** Basic data information and dimensions.

Variable	Type	Units	Dimensions
Patent flows (values)	Numeric	Number	C×S×T×R×Y
Patent stocks (stock values)	Numeric	Number	C×S×T×R×Y

Notes: C = Country, S = Sector (99 2-digit ISIC Rev.4/NACE Rev.2 sectors), T = type (grants or applications), R = patent reference country (applicant’s or inventor’s country of residence), Y =Year (1985 to 2020 - reference date: priority date).

The dataset covers 24 European countries (Austria, Belgium, Bulgaria, Croatia, Czechia, Denmark, Finland, France, Germany, Greece, Hungary, Ireland, Italy, Netherlands, Norway, Poland, Portugal, Romania, Slovak Republic, Slovenia, Spain, Sweden, Switzerland, and the United Kingdom). For each country, up to 99 single-digit sectors are covered, classified according to the International Standard Industrial Classification revision 4 (ISIC Rev.4) that is also consistent with the NACE Rev.2 industry classification and adhere to the 2008 version of the System of National Accounts (SNA). The detailed list is presented in Table 2, using the NACE Rev.2 codes for sections (e.g., A, C, F) and divisions (2-digit classification):

**Table 2:** Sector coverage of the dataset (for each country).

Division (NACE Rev.2, 2-digit)	Section (NACE Rev.2)	Economic Activities
01	A	Crop and animal production, hunting and related service activities
02	A	Forestry and logging
03	A	Fishing and aquaculture
05	B	Mining of coal and lignite
06	B	Extraction of crude petroleum and natural gas
07	B	Mining of metal ores
08	B	Other mining and quarrying
09	B	Mining support service activities
10	C	Manufacture of food products
11	C	Manufacture of beverages
12	C	Manufacture of tobacco products
13	C	Manufacture of textiles
14	C	Manufacture of wearing apparel
15	C	Manufacture of leather and related products of other materials
16	C	Manufacture of wood and of products of wood and cork, except furniture; manufacture of articles of straw and plaiting materials
17	C	Manufacture of paper and paper products
18	C	Printing and reproduction of recorded media
19	C	Manufacture of coke and refined petroleum products
20	C	Manufacture of chemicals and chemical products
21	C	Manufacture of basic pharmaceutical products and pharmaceutical preparations
22	C	Manufacture of rubber and plastic products
23	C	Manufacture of other non-metallic mineral products
24	C	Manufacture of basic metals
25	C	Manufacture of fabricated metal products, except machinery and equipment
26	C	Manufacture of computer, electronic and optical products
27	C	Manufacture of electrical equipment
28	C	Manufacture of machinery and equipment n.e.c.
29	C	Manufacture of motor vehicles, trailers and semi-trailers
30	C	Manufacture of other transport equipment
31	C	Manufacture of furniture
32	C	Other manufacturing
33	C	Repair, maintenance and installation of machinery and equipment
35	D	Electricity, gas, steam and air conditioning supply
36	E	Water collection, treatment and supply
37	E	Sewerage
38	E	Waste collection, treatment and disposal activities; materials recovery
41	F	Construction of buildings
42	F	Civil engineering
43	F	Specialised construction activities
45	G	Wholesale and retail trade and repair of motor vehicles and motorcycles
46	G	Wholesale trade, except of motor vehicles and motorcycles
47	G	Retail trade, except of motor vehicles and motorcycles
49	H	Land transport and transport via pipelines
50	H	Water transport
51	H	Air transport
52	H	Warehousing, storage and support activities for transportation
53	H	Postal and courier activities
55	I	Accommodation
56	I	Food and beverage service activities
58	J	Publishing activities
59	J	Motion picture, video and television programme production, sound recording and music publishing activities
60	J	Programming and broadcasting activities
61	J	Telecommunications
62	J	Computer programming, consultancy and related activities
63	J	Information service activities
64	K	Financial service activities, except insurance and pension funding
65	K	Insurance, reinsurance and pension funding, except compulsory social security
66	K	Activities auxiliary to financial services and insurance activities
68	L	Real estate activities
69	M	Legal and accounting activities
70	M	Activities of head offices; management consultancy activities
71	M	Architectural and engineering activities; technical testing and analysis
72	M	Scientific research and development
73	M	Advertising and market research
74	M	Other professional, scientific and technical activities
78	N	Employment activities
79	N	Travel agency, tour operator and other reservation service and related activities
80	N	Investigation and security activities
81	N	Services to buildings and landscape activities
82	N	Office administrative, office support and other business support activities
84	O	Public administration and defence; compulsory social security
85	P	Education
86	Q	Human health activities
87	Q	Residential care activities
88	Q	Social work activities without accommodation
90	R	Creative, arts and entertainment activities
91	R	Libraries, archives, museums and other cultural activities
92	R	Gambling and betting activities
93	R	Sports activities and amusement and recreation activities
94	S	Activities of membership organisations
95	S	Repair of computers and personal and household goods
96	S	Other personal service activities
97	T	Activities of households as employers of domestic personnel
98	T	Undifferentiated goods- and service-producing activities of private households for own use
99	U	Activities of extraterritorial organisations and bodies

## 4 Experimental Design, Materials and Methods

The dataset was developed based on the following multi-step methodology:

1. Patent applications and grants by year (priority dates), country, reference country and IPC technology field were retrieved in January 2024 from the OECD’s database through the query “Patents by technology” and then filtering for IPC technology fields. It should be noted that the IPC technology fields filter is no longer available in the OECD patent database.^2^
2. Weights for sector correspondence (2-digit level) to IPC technology fields (1-digit level) are retrieved from Lybbert and Zolas [1]. Weights assigning patents to sectors were developed through the ALP method.
3. The weights are utilized to assign patents from IPC fields to NACE Rev.2 sectors.
4. The perpetual inventory method (PIM) is applied to create stocks of patent applications and grants, using a 15% depreciation rate. The initial stock in the starting year (1985) is set as the patent applications/grants of that year.
5. The resulting dataset is consolidated in wide (table-like) and longitudinal formats, and the final data structure is exported to a user-friendly excel file with explanatory notes.
6. Validation actions were implemented across the different steps of the procedure.

**Fig. 1:**
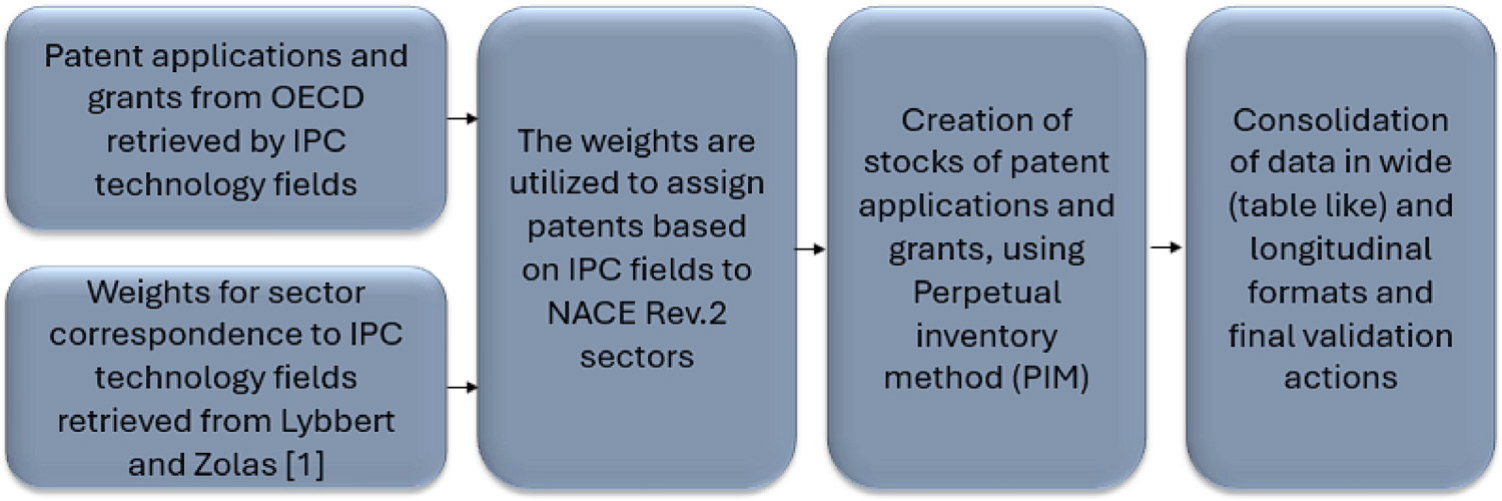
Flowchart of the procedure used.

The above procedure is depicted in the following diagram (Fig. 1):

## Limitations

• The dataset covers only EPO patent applications and grants.
• Due to its source, the dataset is confined to European economies to better capture their innovative output.
• The dataset provides data up to 2020, as OECD no longer provides patent data by IPC technology fields beyond this date.
• While patent data are suitable proxies for technological innovation, they are accompanied by several limitations in terms of capturing the full range of innovation activities at any level of analysis (firm, sector, country).

## Ethics Statement

The authors have read and follow the ethical requirements for publication in Data in Brief and confirm that the current work does not involve human subjects, animal experiments, or any data collected from social media platforms.

## CRediT Author Statement

**Petros Dimas:** Conceptualization, Methodology, Formal Analysis, Data Curation, Writing - Original Draft, Writing - Review & Editing. **Dimitrios Stamopoulos:** Software, Formal Analysis, Data Curation, Writing - Original Draft, Writing - Review & Editing. **Aimilia Protogerou:** Writing - Review & Editing, Project Administration, Supervision.

## Data Availability

Mendeley DataA longitudinal dataset of sector-level patent data for Europe (Original data)

## Footnotes

1 Patent data organized in IPC technology fields are not currently available in OECD’s patent database. Source data in corresponding IPC fields were retrieved on *06-January-2024* and are available upon request for research purposes.

2 At the moment of writing, the latest update of the OECD patent database was implemented in *February-2024*.
